# A Novel Technique for Monitoring Carbonate and Scale Precipitation Using a Batch-Process-Based Hetero-Core Fiber Optic Sensor

**DOI:** 10.3390/s24237580

**Published:** 2024-11-27

**Authors:** Sakurako Satake, Ai Hosoki, Hideki Kuramitz, Akira Ueda

**Affiliations:** 1 Graduate School of Sustainability Studies for Research, University of Toyama, Gofuku 3190, Toyama 930-8555, Japan; 2 Department of Natural and Environmental Sciences, Faculty of Science, Academic Assembly, University of Toyama, Gofuku 3190, Toyama 930-8555, Japan; ahosoki@gipc.akita-u.ac.jp (A.H.); akira@sci.u-toyama.ac.jp (A.U.); 3 Graduate School of Engineering Science, Akita University, Akita 010-8502, Japan

**Keywords:** fiber optic sensor, batch process method, scale, CO_2_ mineralization, hetero-core fiber optic

## Abstract

Techniques for monitoring calcium carbonate and silica deposits (scale) in geothermal power plants and hot spring facilities using fiber optic sensors have already been reported. These sensors continuously measure changes in light transmittance with a detector and, when applied to field tests, require the installation of a power supply and sensor monitoring equipment. However, on some sites, a power supply may not be available, or a specialist skilled in handling scale sensors is required. To overcome this problem, we have developed a method for evaluating scale formation that is based on a batch process that can be used by anyone. In brief, this method involves depositing scale on a section of the optical fiber sensor and then fusing this section to the optical fiber and measuring it. Using this sensor, a technician in the field can simply place the sensor in the desired location, collect the samples at any given time, and send them to the laboratory to measure their transmittance. This simple and easy method was achieved by using a hetero-core type of fiber optic. This evaluation method can measure with the same sensitivity as conventional real-time methods, while its transmittance response for the sensor corresponds to the saturation index (SI) changes in the scale components in the solution due to increases in temperature and concentration. In the field of carbon dioxide capture and storage (CCS), this evaluation method can be used to quantitatively measure the formation of carbonate minerals, and it can also be used as an indicator for determining the conditions for CO_2_ mineral fixation, as well as in experiments using batch-type autoclaves in laboratory testing. It is also expected to be used in geothermal power plants as a method for evaluating scale formation, such as that of amorphous silica, and to protect against agents that hinder stable operation.

## 1. Introduction

**Figure 1:**
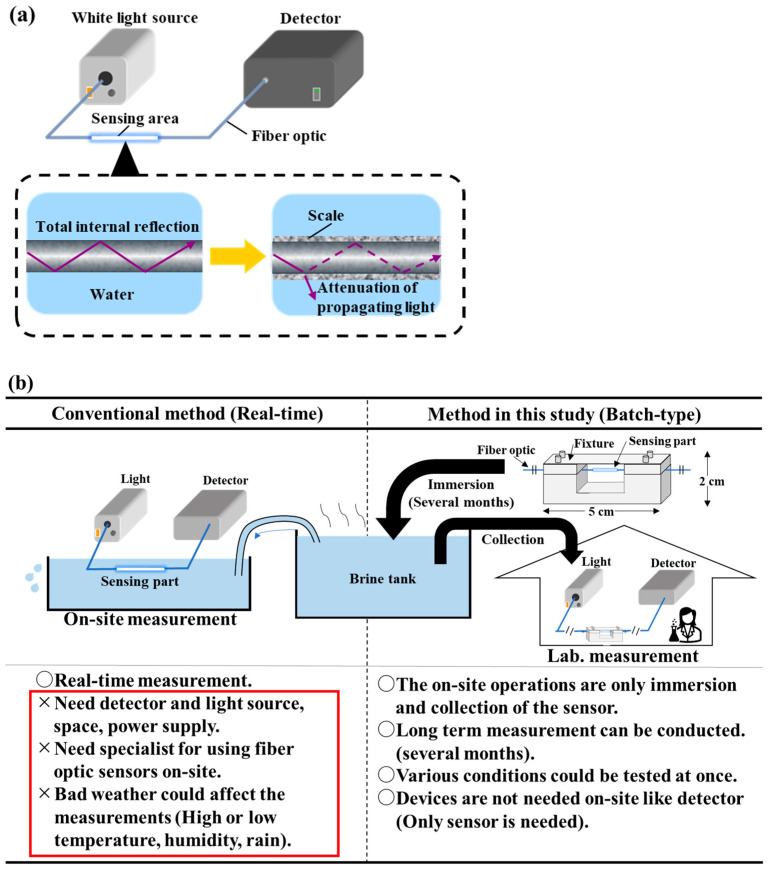
(**a**) Scale sensor conceptual diagram. Transmittance decreases due to the deposition of scale on the sensor. (**b**) Conceptual diagram of the batch-type method. In the conventional method (real-time), there are some problems. For example, it necessitates devices like a detector and a specialist for using sensors on-site, and it could be affected by bad weather. The batch-type method could solve these problems. This method allows for the easy on-site operation and does not require operations by a specialist, and implementation of a number of test conditions.

In geothermal power plants, geothermal scale, such as carbonate and silica compounds, is formed in the piping due to the supersaturation of inorganic salts in the geothermal fluid because of pressure and temperature changes. This accumulation of scale is a problem because it interferes with the stable operation of geothermal power plants. Scale control measures include pH adjustments, high-temperature reinjection, and the removal of excess silica from geothermal brine [1,2,3,4,5,6]. In these scale-prevention technologies, a variety of methods have been used to evaluate test results, including visual inspection, the evaluation of weight changes in metal pieces immersed in geothermal water, and the examination of changes in the water flow rate using column devices that simulate a formation [6,7,8,9,10,11]. However, these evaluation methods are time-consuming, costly, and inconvenient. There is therefore a need to develop a method that can evaluate scale precipitation faster, with greater ease and accuracy. So, our laboratory has developed several fiber optic “scale sensors” that can evaluate scale formation on-site in a short amount of time and in real time by taking advantage of the characteristics of optical fibers, such as their pressure and heat resistance, which make them suitable for measurements in geothermal fluids (real-time-type methods) [12,13,14,15,16,17]. In this method, one end of the fiber optic sensor is connected to a light source and the other end is connected to a spectrometer to measure scale generation while monitoring it in real time (Figure 1a). In this sensing system, inorganic salts with a refractive index higher than that of the sensing part adhere to the sensing part, preventing the total reflection of the light propagating into the sensor and attenuating the amount of light propagation, thus allowing for the evaluation of scale generation. To confirm the effectiveness of the sensor, various tests were also conducted in geothermal water utilization facilities like the *Sumikawa* geothermal power plant [12,13,15,17]. This method is suitable for short-term measurements, but technically struggles to measure scale accumulation in the long term and in high-temperature and high-pressure environments in real time. In addition, current methods require specialized knowledge to manipulate the sensors, and technicians at geothermal power plants or other sites cannot use them. Also, when applied in the field, power supplies for devices like PCs are also needed, but at some sites, these power supplies may not be available. Since our scale sensor is intended for use in geothermal water utilization facilities and at other sites, it is necessary to have a measurement method that can be used by anyone, that can be easily used at sites where it is difficult to secure a power source and space for measurement, and that can be used even with sudden weather changes (high temperatures, low temperatures, humidity, and heat).

In this study, we propose a method based on batch processes that anyone can use to easily evaluate scale formation in geothermal brine on-site (batch-type method, Figure 1b). In this method, a section of an optical fiber sensor is immersed in geothermal brine, and, after a certain amount of time, the optical fiber section is connected to a measuring instrument to evaluate the inorganic salt precipitation collected based on the propagating light intensity of the optical fiber before and after the reaction. The interval between the immersion of the optical fiber section in the geothermal fluid and the subsequent laboratory measurement can be specified at the user’s discretion, with an interval of months. This novel measurement method reduces the amount of on-site work and measuring equipment required, is not affected by weather conditions, and is a simple measurement method that solves the problems of real-time methods. In this article, we verified whether it can be manufactured stably, whether it has the same sensitivity and responses as a conventional real-time method, and whether it can be used at high temperatures.

**Figure 2:**
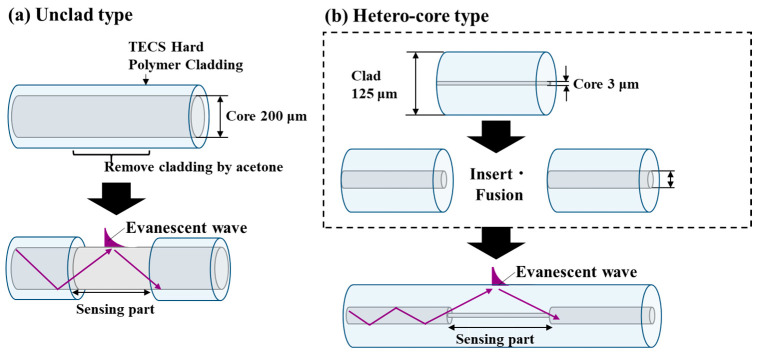
(**a**) Unclad-type fiber optic sensor. In this structure, the sensing area is made by removing cladding to expose the core part. (**b**) Hetero-core-type fiber optic sensor. The sensor is made from two different types of fiber optics in terms of core diameter.

Moreover, the sensor’s structure was also investigated. Until now, fiber optic scale sensors have been unclad fiber optic sensors, fabricated by removing the coating and cladding from a 200 µm core diameter step-index multimode optic fiber to expose its core (Figure 2a; hereinafter referred to as an “unclad sensor”) [12,13,14,15,16,17]. In this study, in terms of batch-type methods, the hetero-core fiber optic sensor was investigated in addition to the unclad-type sensor. The hetero-core sensor was developed by Watanabe et al. (Figure 2b; hereinafter referred to as the hetero-core sensor) [18,19,20,21,22]. This hetero-core fiber sensor has an easily fabricated simple structure with no need for cladding removal and can be fabricated by using the optical fibers used for general telecommunications (with core diameters of 50 μm and 62.5 μm), which are extremely flexible and easier to handle. In this structure, the cladding surface can work as a sensing region because transmitted light is largely leaked into the cladding layer due to the core diameter difference between the transmission and inserted fibers. The hetero-core sensor has been applied as a sensor to measure acidity, humidity, liquid concentration, and hydrogen gas [18,19]. Conventional unclad-type fiber optic scale sensors have large core diameters, such as 200 μm, which make them inflexible, but a hetero-core sensor could be thinner and easier to handle. In this study, we evaluated the difference between hetero-core and unclad sensors, and which sensor is better for the batch-type method.

The batch-type method needs to be used efficiently in settings other than geothermal facilities. Currently, carbon dioxide capture and storage (CCS) technology is attracting attention as a means of preventing global warming. In the CCS field, supercritical CO_2_ is injected into sedimentary formations and oil reservoirs at temperatures below 100 °C [23,24,25]. During this process, a proportion of the injected CO_2_ will also be immobilized underground by CO_2_–H_2_O–rock interaction, forming carbonate minerals. The injection of CO_2_ results in the dissolution of groundwater and geothermal water, leading to an acidic condition. This promotes the dissolution of rocks, with divalent cations such as Ca^2+^ and Mg^2+^ combining with CO_3_^2−^ to form carbonate minerals, including calcium carbonate, which are fixed underground as secondary minerals [26]. In Iceland, the Carbfix project involves injecting CO_2_–water into a geothermal area, 72% of which is estimated to be mineralized to calcite [27]. In this innovative technology, determining efficient and effective CO_2_ concentrations and fixation rates/mechanisms for carbonate mineralization plays an important role in promoting decarbonization. In order to evaluate CO_2_ mineralization, various batch-type tests on CO_2_–H_2_O–rock interactions have been conducted [27,28,29,30]. Gysi et al. investigated the reaction of basalt with CO2-dissolved water and observed the formation of secondary minerals, including clay minerals, at an early stage of the reaction. Subsequently, carbonate minerals were formed after the formation of clay minerals [28]. In addition, Voigt et al. investigated the reaction of mid-ocean ridge basalt (MORB) glass and North Atlantic seawater filled with 2.5 bar pCO_2_. Their findings indicate that 20% of the CO_2_ was mineralized within 5 months [30]. The course of the CO_2_–H_2_O–rock reaction was determined by changes in the solution composition in the experimental solution and the rock surface after the experiment was completed. The reaction rate is often estimated using simulation software based on changes in the solution composition in the experimental solution and changes in the surface of the rock after the experiment. However, there are few examples of quantitative monitoring of the reaction rate. Several methods have been developed for the real-time monitoring of mineral formation phenomena, such as the formation of calcium carbonate (CaCO_3_) [31,32,33,34]. Heine et al. have employed the use of satellite images to monitor the formation of calcium carbonate in lakes in real time, utilizing the inherent color differences present in these images [31]. Recently, Zotzmann et al. have monitored the precipitation of barite in the laboratory using optical fibers under conditions of up to 5 M NaCl at 150 °C [32]. In addition, research into the in situ monitoring of the hydration of calcium silicate minerals in cement using fiber optic Raman probes has been carried out [33]. An electrochemical quartz crystal microbalance (EQCM) has been adapted to monitor CaCO_3_ formation, and it has been found that the application of a voltage enhances the amount of CaCO_3_ deposition [34]. These are very important techniques for elucidating the formation mechanism, such as the rate of CaCO_3_ scale formation. Furthermore, in contrast to real-time measurement methods, the batch-type method proposed in this study eliminates the need to connect equipment at the measurement site. Subsequently, the entire apparatus can be linked together once the examination is complete. This allows multiple points to be measured simultaneously and different test conditions to be investigated, providing a rapid assessment of scale formation mechanisms. Furthermore, the batch-type method can be easily incorporated into ongoing CO_2_–H_2_O–rock reaction experiments without requiring significant alterations to their existing configurations. Consequently, it can also be applied to the evaluation of the generation rates of secondary minerals, such as carbonate minerals. Additionally, as there is no requirement to connect to the measurement instruments during the experiment, it is possible to conduct a multitude of test conditions simultaneously, facilitating rapid and straightforward investigations into reaction behavior. Furthermore, the application of this methodology to ongoing research on the mineralization of CO_2_ will facilitate a more comprehensive evaluation of the reaction mechanism.

## 2. Materials and Methods

### 2.1. Chemical Reagents and Materials

Sodium hydrogen carbonate and calcium chloride were purchased from FUJIFILM Wako Pure Chemicals Industries (Osaka, Japan). All reagents were of analytical grade, and solutions of these compounds were prepared using distilled water.

### 2.2. Sensor Fabrication

For the unclad fiber optic sensor showed in Figure 2a, a step-index multimode optical fiber (FT200EMT; Thorlabs, Newton, NJ, USA) with a 200 μm diameter fused silica core was used. The refractive index of the optic fiber was 1.451, and it was surrounded by TEQSTM polymer cladding with a refractive index of 1.392 at 1020 nm. The fiber cladding was carefully removed from the middle of the fiber by rubbing and using acetone to expose the fiber core.

A hetero-core fiber optic scale sensor was fabricated by inserting and fusing a 15 mm single-mode fiber optic (SMF) with a 3.1 µm core diameter into a multimode fiber (MMF) with a 50 µm core diameter using a fiber cleaver (CT-32, Fujikura Ltd., Tokyo, Japan) and a thermal fusion splicer (FSM-100P+, Fujikura Ltd.) (Figure 2b). This fabrication method is a relatively well-established technology used in the maintenance and operation of fiber optic communication networks and serves as a simpler method.

### 2.3. Laboratory Study

**Figure 3:**
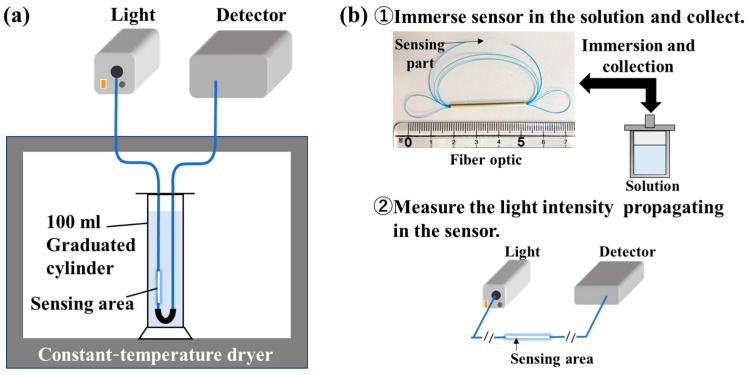
(**a**) Real-time-type method. The fiber optic sensor is connected to a light source and detector throughout the measurement period. (**b**) Batch-type method. First, the optic fiber is immersed in the test solution. After a certain period of time, it is collected and subsequently connected for evaluation.

For the real-time method, the optic fiber was passed through a metal U-shaped tube so that the fiber optic sensing was perpendicular to the ground and immersed in a 100 mL graduated cylinder containing a mixture (Figure 3a) with equal amounts of 5 mM, 10 mM, 50 mM, and 100 mM in NaHCO_3_ and CaCl_2_ solutions. Both ends of the sensor were fused to patch cables (Patch cable, Thorlabs) and connected to a light source (Tungsten Halogen Light Sources, Ocean Insight) at one end and a spectrometer (MV-3000 series, JASCO Corp., Tokyo, Japan) at the opposite end to continuously measure the change in transmittance response. The measuring cylinder and sensor portion were placed in a constant-temperature dryer maintained at 25 °C and 95 °C for 3 h.

For the batch-type evaluation method, a piece of the fabricated fiber optic sensor was placed in a Teflon cup (Figure 3b). Then, the test solution was added. This solution has the same formula as that used in the real-time method. Next, the Teflon cup was placed in a high-temperature, high-pressure container made of SUS316 (nozzle-type stainless steel inner tube, Sanai-Kagaku, Nagoya, Japan) and reacted in a constant-temperature dryer at 25 °C to 150 °C. After the reaction was completed, the high-temperature, high-pressure container was removed from the constant-temperature dryer and cooled with water, and the sensor was collected. The collected sensor was then fused to a patch cord using a fusion tool, and light intensity was measured by connecting it to a light source and spectrometer. During fusion splicing, the amount of light loss was inspected by the fusion splicer, and if a loss of 0.2 dB or more occurred, it was re-fused to keep the loss within 0.2 dB. In addition, to prevent loss due to the bending of the sensor portion during measurement, the sensor was straightened.

In those methods, transmittance (%) was calculated at a wavelength of 560 nm as

$$Transmittance\left(\%\right)=\frac{I_{t}}{I_{0}}\times 100$$

where *I*_0_ is the initial transmitted light intensity and *I_t_* is the transmitted light intensity by time after immersion, respectively.

### 2.4. Analysis

The sensor surfaces before and after were observed by scanning electron microscopy (SEM) equipped with energy dispersive X-ray spectroscopy (JCM7000 + JED2300, JEOL Ltd., Tokyo, Japan). After the transmittance evaluation of batch-type experiments, sensors were analyzed to observe mineral shapes by SEM.

## 3. Results and Discussion

### 3.1. Comparison by Sensor Structure

**Figure 4:**
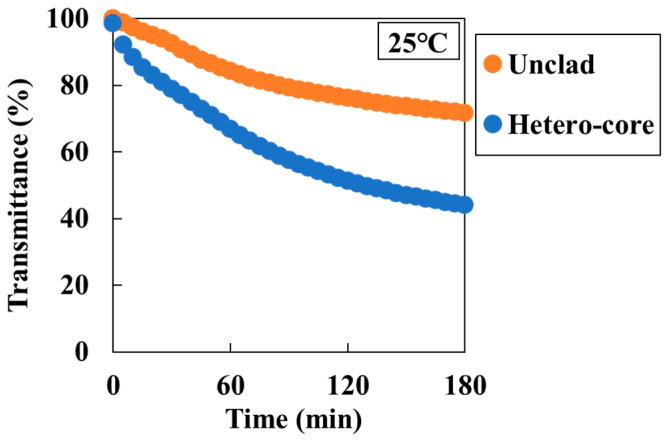
Transmittance response of the sensor for 50 mM (Ca^2+^) solution in 180 min by sensor structures. Orange plots show the transmittance changes in the unclad sensor; blue plots show the transmittance changes in the hetero-core sensor.

In this part, a comparison of the differences in sensitivity between sensor structures was conducted in order to ascertain which sensor structure is most suitable for the new batch evaluation method. Unclad and hetero-core sensors were used to measure the sensor response at 25 °C in a mixture of aqueous NaHCO_3_ (100 mM) and CaCl_2_ (100 mM) solutions in which CaCO_3_ precipitates. The sensing area of both sensors was standardized to 15 mm. For the unclad sensor, transmittance decreased to approximately 70% after 3 h (Figure 4). On the other hand, the hetero-core sensor’s transmittance decreased to approximately 40%, accompanied by heightened sensitivity in comparison to the unclad sensor. The sensitivity depends on the number of reflections within the waveguide [12,35] and can be improved even in the unclad sensor if the core diameter can be further reduced. However, in optic fibers with smaller diameters, the cladding is made of silica material, which cannot be easily removed. Therefore, it becomes difficult to fabricate reproducible sensors. In this study, a hetero-core optic fiber, which has a shorter sensing length and higher sensitivity and is good at handling, was used and was concluded to be good for the batch-type method.

### 3.2. Reproducibility of Fiber Optic Sensors

The batch-type method requires reproducibility with respect to sensor fabrication and the connection of the sensors to a light source and spectrometer in order to facilitate a comparison of the light intensity of the optical fiber sensors prior to and following the formation of the scale. Therefore, the intensity of light propagating between individual sensors must be the same. Previously, it was usual to manually polish the sensor end faces or connect them to a terminator (SMA905 and BFT1, Thorlabs), which resulted in errors when connecting them to the measurement device. In this research, instead of manual operation, a thermal fusion splicer was used to mechanically connect the sensor to the measurement device, thereby enabling application to batch-type sensors. So, it can be reasonably deduced that the light intensity is comparable. The reproducibility of measurements for fiber optic sensors was investigated, with a particular focus on unclad and hetero-core sensors. For each sensor, three optic fibers were fabricated, and the reproducibility of measurements was evaluated based on the propagation error in the fiber optics, which was determined by the light intensity. Consequently, it was possible to measure the light intensity with a coefficient variation of 0.06 for the unclad type. Additionally, the hetero-core type could be quantified with a coefficient variation of 0.03 (Table 1). The fabrication process involves fusing the sensor to the patch cord when connecting the sensor to the light source and spectrometer. It can be concluded that the sensor can be used as an evaluation method, regardless of whether it is of the hetero-core or unclad type.

### 3.3. Real-Time and Batch-Type Methods

**Figure 5:**
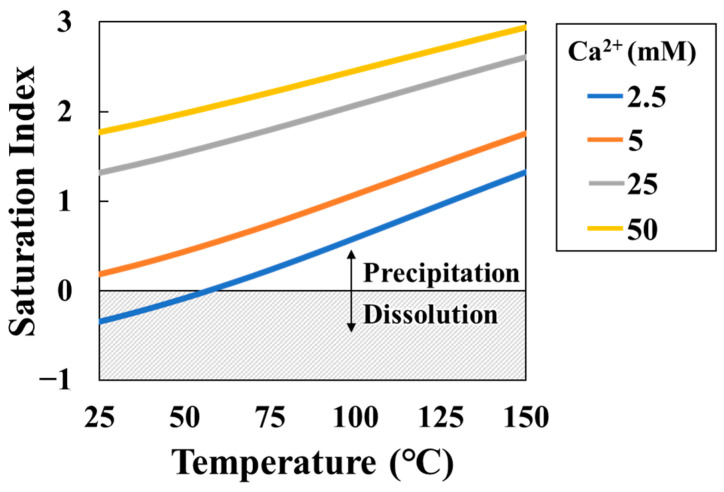
Saturation index (SI) changes as functions of the temperature and solution concentration (mixture of same concentration of NaHCO_3_ and CaCl_2_) (blue line, 2.5 mM (Ca^2+^); orange line, 5.0 mM (Ca^2+^); gray line, 25 mM (Ca^2+^); yellow line, 50 mM (Ca^2+^)).

In order to evaluate the effectiveness of the batch-type method, a comparison was conducted between the results obtained from the real-time-type and batch-type approaches. Before the experiments, the geochemical calculation code PHREEQC was used to calculate the saturation index (SI) variation in calcite with various Ca^2+^ concentrations and reaction temperatures in NaHCO_3_ and CaCl_2_ solutions (Figure 5) [36]. The SI exhibits an increase in accordance with both temperature and Ca^2+^ concentration. In a solution with a Ca^2+^ concentration of 2.5 mM, the SI is unsaturated at −0.34 (25 °C) but becomes supersaturated at 0.52 at 95 °C. Therefore, as the temperature and Ca^2+^ concentration increase, the amount of CaCO_3_ produced increases. Consequently, the resulting transmittance response of the sensor is expected to be greater.

**Figure 6:**
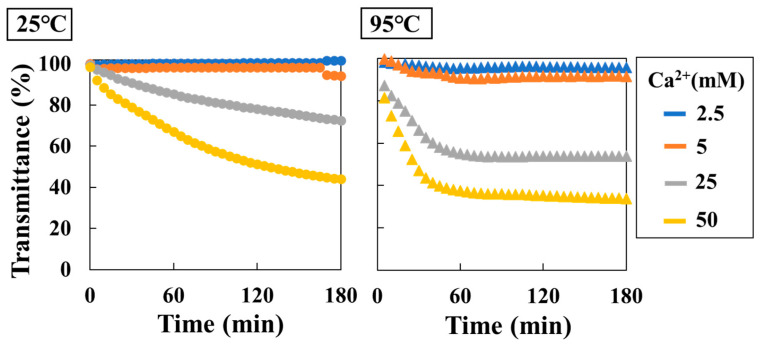
Transmittance changes for CaCO_3_ precipitation in 180 min (**left**, 25 °C; **right**, 95 °C; blue plots, 2.5 mM (Ca^2+^); orange plots, 5.0 mM (Ca^2+^); gray plots, 25 mM (Ca^2+^); yellow plots, 50 mM (Ca^2+^)).

The real-time method was performed using a hetero-core sensor at Ca^2+^ solution concentrations ranging from 2.5 mM to 50 mM at temperatures of 25 °C and 95 °C (Figure 6). A decrease in transmittance was observed with an increase in Ca^2+^ concentration at 25 °C and 95 °C. In particular, at a Ca^2+^ concentration of 2.5 mM, no transmittance response in the sensor was observed at 25 °C due to undersaturation. Meanwhile, at 95 °C, supersaturation occurred, and a decrease in transmittance of approximately 10% was observed. Furthermore, as the degree of supersaturation increased, the initial rate of transmittance that had decreased (%/min) also increased (Table 2).

**Figure 7:**
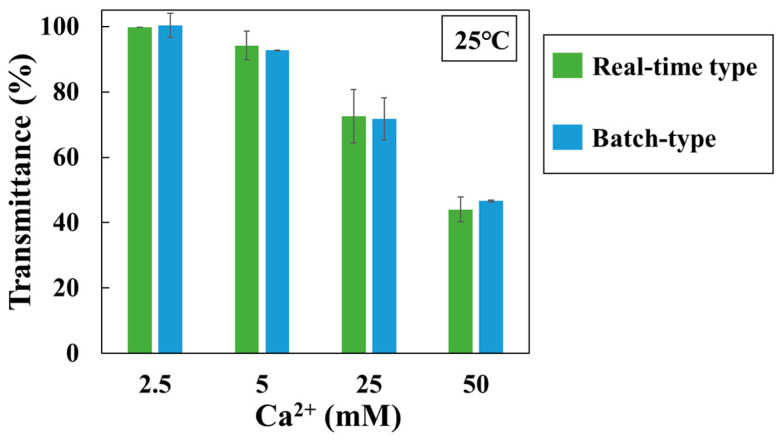
Transmittance changes for different concentration of Ca^2+^ in 180 min by two methods (25 °C). The green bar is the change for the real-time type method; the blue bar is the change for the batch-type method. Each error bar shows standard deviation.

The batch-type method was also performed using a hetero-core. The light intensity transmitted through the optical fiber after 3 h of immersion in the solution at 25 °C was compared (Figure 7). Between both methods, with an increase in Ca^2+^ concentration and SI, the transmittance (%) decreased. The discrepancy in measurements between the two measurement methods was within 5%. According to the results, the difference between the two methods was within the acceptable error range, indicating that the batch-type method was effective.

**Figure 8:**
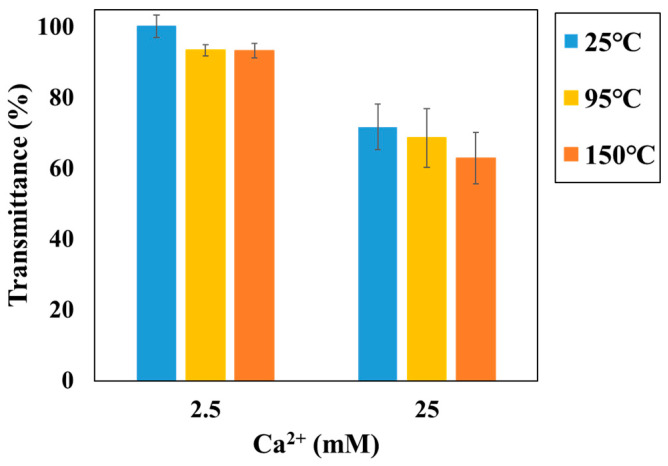
Transmittance change at different temperatures and Ca^2+^ concentrations by the batch-type sensor (blue bar, 25 °C; yellow bar, 95 °C; orange bar, 150 °C; error bar, standard deviation).

**Figure 9:**
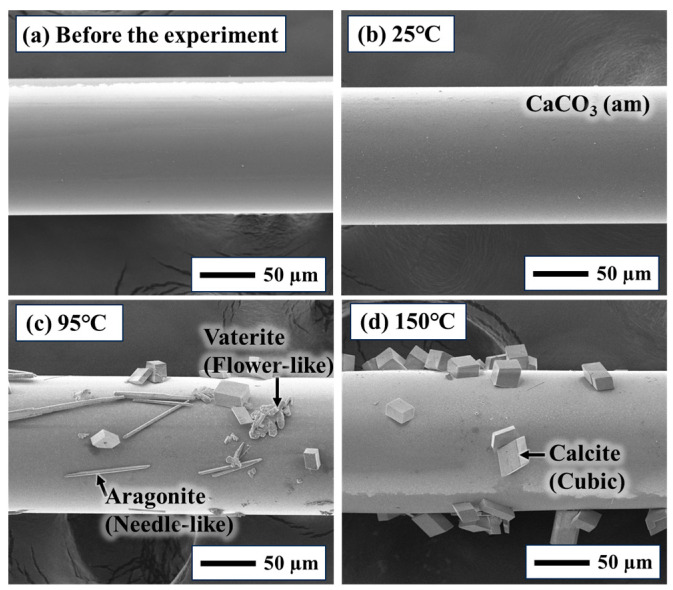
SEM images of the sensor surface before and after the measurement in 5 mM Ca^2+^ solution ((**a**) before the experiment, (**b**) after the experiment at 25 °C, (**c**) 95 °C, and (**d**) 150 °C). Various polymorphs of CaCO_3_ were observed: needle-shaped aragonite, flower-shaped vaterite, cubic-shaped calcite, and amorphas CaCO_3_.

The results obtained using the batch-type method in different temperature (25–150 °C) are shown in Figure 8 (Reaction time is 3 h). A decrease in the transmittance response of the sensor was observed with an increase in Ca^2+^ concentration and temperature. Thus, similar to the SI calculation results in Figure 5, calcium carbonate is easily formed at high temperatures and concentrations, indicating that the batch-type method can be used even under high-temperature conditions. In high-temperature conditions, the batch-type method facilitates the measurement of transmittance changes in differences in SI. The sensor surface was observed by SEM before and after the experiments (Figure 9). Okazaki et al. discussed the relationship between the transmittance response of the sensor and the amount of scale deposition on the sensor surface and concluded that there is a correlation between the sensor response and the scale coverage on the sensor surface [12]. Figure 9 shows the results for the sensor surface image, which indicates an increase in scale coverage on the optical fiber with increasing temperature for the same concentration of the test solution. Furthermore, the CaCO_3_ precipitates formed on the sensor surface exhibited different crystal structures depending on the temperature (25 °C, amorphous CaCO_3_; 95 °C, various polymorphs were observed including cubic calcite, needle-like aragonite and flower-like vaterite; 150 °C, most of the polymorphs were calcite). In accordance with the findings by Hashimoto et al., the most stable polymorph of CaCO_3_ crystal is calcite. The results of this study confirm the phase change from crystal structures, such as aragonite and vaterite to calcite under high-temperature conditions [37]. This result also supports the fact that the batch measurement technique can clearly distinguish the difference in response under high-temperature conditions. As the sensor response increased with increasing SI, it seems that the response observed in the results of this study was also regulated by the increase in CaCO_3_ precipitation with increasing SI.

## 4. Conclusions

The objective of this study was to develop a technique for monitoring scale formation in geothermal brine, applying a batch-process-based fiber optic sensor to enhance the convenience of the usage of this technique in a geothermal power plant and in carbon dioxide (CO_2_) mineralization fields. Conventional real-time monitoring techniques present significant challenges in field applications, necessitating a power supply, specialized expertise, and vulnerability to environmental factors such as weather. In contrast, the batch-type sensor method offers a straightforward, user-friendly approach that maintains high reproducibility and exhibits a sensitivity comparable to that of real-time methods.

The experiments demonstrated that the hetero-core sensor exhibited greater sensitivity than the traditional unclad sensor. Furthermore, the reproducibility of the proposed method was evaluated, demonstrating that the hetero-core sensor yielded consistent and reliable measurements with minimal variation. A comparison of the batch-type and real-time methods revealed a strong correlation, thereby further validating the effectiveness of the batch-type sensor in a range of environmental conditions.

The batch-type measurement method has the potential to be employed not only for the evaluation of scale formation in geothermal power plants but also for the quantitative assessment of carbonate mineralization in CO_2_ sequestration processes. Since this method does not require connection to measurement equipment during the experiment, more test conditions can be performed at once, allowing the reaction behavior to be investigated quickly and easily. Furthermore, if adapted to existing CO_2_ mineralization study, it should contribute to a more detailed evaluation of the reaction mechanism. In light of the pivotal role of CO_2_ mineralization in mitigating climate change, this innovative sensor method has the potential to become a pivotal tool in advancing carbon reduction strategies. The findings indicate that the batch-process-based hetero-core fiber optic sensor will play an instrumental role in ensuring the stability of geothermal operations and enhancing the evaluation of CO_2_ mineralization.

## Figures and Tables

**Table 1:** Reproducibility of sensor fabrication for the unclad sensor and hetero-core sensor.

	Wavelength (nm)	Number of Samples	Average	Standard Deviation	Coefficient of Variation
Unclad	560	3	46947	2904	0.06
Hetero-core	560	3	48797	1273	0.03

**Table 2:** Initial response time up to 30 min (%/min) for the hetero-core sensor in different Ca^2+^ concentration solutions (2.5–50 mM (Ca^2+^)).

Reaction Temperature (°C)	Ca^2+^ (mM)			
	2.5	5	25	50
25 °C	-	0.07	0.29	0.66
95 °C	0.20	0.30	1.22	1.94

## Data Availability

The data are contained within the article.
